# Intestinal, extra-intestinal and systemic sequelae of *Toxoplasma gondii* induced acute ileitis in mice harboring a human gut microbiota

**DOI:** 10.1371/journal.pone.0176144

**Published:** 2017-04-17

**Authors:** Eliane von Klitzing, Ira Ekmekciu, Anja A. Kühl, Stefan Bereswill, Markus M. Heimesaat

**Affiliations:** 1 Department of Microbiology and Hygiene, Charité - University Medicine Berlin, Berlin, Germany; 2 Department of Medicine I for Gastroenterology, Infectious Diseases and Rheumatology / Research Center ImmunoSciences (RCIS), Charité – University Medicine Berlin, Berlin, Germany; NIH, UNITED STATES

## Abstract

**Background:**

Within seven days following peroral high dose infection with *Toxoplasma gondii* susceptible conventionally colonized mice develop acute ileitis due to an underlying T helper cell (Th) -1 type immunopathology. We here addressed whether mice harboring a human intestinal microbiota developed intestinal, extra-intestinal and systemic sequelae upon ileitis induction.

**Methodology/Principal findings:**

Secondary abiotic mice were generated by broad-spectrum antibiotic treatment and associated with a complex human intestinal microbiota following peroral fecal microbiota transplantation. Within three weeks the human microbiota had stably established in the murine intestinal tract as assessed by quantitative cultural and culture-independent (i.e. molecular 16S rRNA based) methods. At day 7 post infection (p.i.) with 50 cysts of *T*. *gondii* strain ME49 by gavage human microbiota associated (hma) mice displayed severe clinical, macroscopic and microscopic sequelae indicating acute ileitis. In diseased hma mice increased numbers of innate and adaptive immune cells within the ileal mucosa and lamina propria and elevated intestinal secretion of pro-inflammatory mediators including IFN-γ, IL-12 and nitric oxide could be observed at day 7 p.i. Ileitis development was accompanied by substantial shifts in intestinal microbiota composition of hma mice characterized by elevated total bacterial loads and increased numbers of intestinal Gram-negative commensals such as enterobacteria and *Bacteroides / Prevotella* species overgrowing the small and large intestinal lumen. Furthermore, viable bacteria translocated from the inflamed ileum to extra-intestinal including systemic compartments. Notably, pro-inflammatory immune responses were not restricted to the intestinal tract as indicated by increased pro-inflammatory cytokine secretion in extra-intestinal (i.e. liver and kidney) and systemic compartments including spleen and serum.

**Conclusion/Significance:**

With respect to the intestinal microbiota composition “humanized” mice display acute ileitis following peroral high dose *T*. *gondii* infection. Thus, hma mice constitute a suitable model to further dissect the interactions between pathogens, human microbiota and vertebrate host immunity during acute intestinal inflammation.

## Introduction

Within one week following peroral infection with a high dose (i.e. 50 to 100 cysts) of the intracellular parasite *Toxoplasma gondii* strain ME49 susceptible mice develop severe ileitis and succumb to infection [1–3]. Disease develops due to a classical T helper cell (Th1)-type hyper-inflammatory immune response that is characterized by a CD4+ T lymphocyte driven excessive secretion of pro-inflammatory mediators such as TNF, IFN-γ, nitric oxide (NO) and IL-12, whereas counter-regulatory measures include IL-10 production [3–7]. Ileitis development has been shown to be highly microbiota dependent [8], given that secondary abiotic mice with a virtually depleted intestinal microbiota were unaffected following *T*. *gondii* infection, whereas upon reconstitution with the murine intestinal microbiota mice were suffering from overt disease [9]. Acute ileitis was further accompanied by a marked shift in the intestinal microbiota composition towards an overgrowth of the inflamed ileal lumen with commensals such as enterobacteria and *Bacteroides / Prevotella* spp. [3, 9, 10]. Toll-like receptor (TLR) -4 dependent signaling of lipopolysaccharide (LPS) derived from the overgrowing Gram-negative bacterial species such as *E*. *coli* further perpetuate the fatal immunopathological process [11]. Overall, the high dose *T*. *gondii* infection model resembles key features of inflammatory bowel diseases (IBD) in humans such as Crohn’s disease during the acute stage [3, 9, 12].

Given the importance of the host specific intestinal microbiota in susceptibility towards distinct immunopathological diseases, their onset, progression and outcome [8, 13, 14], we generated with respect to their microbiota “humanized” mice in order to mimic human microbiota conditions for the investigations of the molecular mechanisms underlying pathogen-commensal bacterial-host interactions. To address this, conventional mice were subjected to broad-spectrum antibiotic treatment in order to virtually deplete the commensal intestinal microbiota [8, 9]. Secondary abiotic mice were then reassociated with a complex human intestinal microbiota by fecal microbiota transplantation (FMT) that could stably establish within the murine host for at least six weeks [14]. In the present study we applied the human microbiota associated (hma) mouse model to unravel the triangle relationship between pathogen, human intestinal microbiota ecology and host immunity in acute *T*. *gondii* induced ileitis.

## Material and methods

### Ethical statement

All animal experiments were conducted according to the European Guidelines for animal welfare (2010/63/EU) with approval of the commission for animal experiments headed by the “Landesamt für Gesundheit und Soziales” (LaGeSo, Berlin; registration number G0184/12). Animal welfare was monitored twice daily by assessment of clinical conditions and weight loss of mice. Mice suffering from weight loss >20% were euthanized by isoflurane treatment (Abott, Germany) in accordance with the guidelines of the local authorities (“human end points”).

### Generation of human microbiota associated mice

Female C57BL/6j mice were bred under specific pathogen-free conditions in the Forschungseinrichtungen für Experimentelle Medizin (Charité—University Medicine, Berlin, Germany). Secondary abiotic mice with a virtually depleted microbiota were generated as described previously [9]. In brief, eight weeks old mice were transferred into sterile cages and subjected to a broad-spectrum antibiotic treatment for eight weeks by adding ampicillin plus sulbactam (1 g/L; Ratiopharm, Germany), vancomycin (500 mg/L; Cell Pharm, Germany), ciprofloxacin (200 mg/L; Bayer Vital, Germany), imipenem (250 mg/L; MSD, Germany) and metronidazole (1 g/L; Fresenius, Germany) to the drinking water (*ad libitum*).

Three days prior reassociation of secondary abiotic mice with a complex human intestinal microbiota, antibiotic cocktail was replaced by autoclaved tap water (*ad libitum*). Fresh fecal samples free of enteropathogenic bacteria, viruses and parasites were collected from five individual healthy volunteers, dissolved in sterile phosphate buffered saline (PBS; Gibco, life technologies, UK)), aliquoted and stored at -80°C as described earlier [14]. Immediately before reconstitution experiments, individual fecal aliquots were thawed, pooled, and the main bacterial communities within the donor suspension quantitatively assessed by culture and molecular methods as stated previously [14]. To generate human intestinal microbiota associated (hma) mice, secondary abiotic animals were subjected to peroral fecal transplantations with 0.3 mL of the donor suspension by gavage on three consecutive days. Bacterial groups varied less than 0.5 logarithmic orders of magnitude between independent experiments. To assure proper establishment of the human microbiota in the murine host, mice were kept for three weeks until ileitis induction. Immediately before peroral *T*. *gondii* infection individual fecal samples were collected for quantitative cultural and molecular analyses of main intestinal bacterial communities as described elsewhere [9, 11, 14, 15].

### *Toxoplasma gondii* infection and clinical conditions

In order to induce acute ileitis, hma were perorally subjected to high-dose *T*. *gondii* ME49 strain infection (i.e. with 50 cysts) by gavage as described previously [9, 11, 16, 17]. Body weights as well as macroscopic and / or microscopic abundances of fecal blood were assessed in individual mice on a daily basis by the Guajac method using Haemoccult (Beckman Coulter/ PCD, Germany). Noninfected hma mice served as negative controls.

### Sampling procedures

Mice were sacrificed seven days after ileitis induction by isoflurane treatment (Abott, Germany). Cardiac blood, tissue samples from spleen, liver, lung, kidney, mesenteric lymph nodes (MLN), ileum and colon were removed under sterile conditions. Colonic and ileal samples from each mouse were collected in parallel for microbiological, immunological, immunohistochemical and histopathological analyses.

### Small intestinal lengths and histopathological scores

Small intestinal lengths were determined by measuring the distance from the duodenum leaving the stomach to the ileal-caecal transition. *Ex vivo* biopsies derived from the terminal ileum were immediately fixed in 5% formalin and embedded in paraffin. Sections (5 μm) were stained with hematoxylin and eosin (H&E) and subjected to a standardized histopathological scoring system ranging from 0 to 6 as described earlier [9, 11].

### Immunohistochemistry

Five μm thin paraffin sections of ileal *ex vivo* biopsies were used for *in situ* immunohistochemical analyses as described previously [15, 18, 19]. Primary antibodies against cleaved caspase-3 (Asp175, Cell Signaling, Boston, MA, USA, 1:200), CD3 (Polyclonal rabbit anti human, DAKO, Denmark; 1:10), FOXP3 (FJK-165, eBioscience, Germany; 1:100), B220 (eBioscience; 1:200) and F4/80 (biot. Clone BM 8 rat anti-mouse, Life Technologies, USA; 1:100) were used to assess apoptotic cells, T lymphocytes, regulatory T cells (Treg), B lymphocytes and macrophages / monocytes, respectively. The average number of positively stained cells within at least six high power fields (HPF, 0.287 mm^2^; 400 x magnification) were determined by an independent and blinded investigator.

### Cultural survey of the human donor suspension, intestinal microbiota and bacterial translocation

For comprehensive quantitative survey of the microbiota composition in fecal human donor suspensions and intestinal (i.e. ileal and colonic) luminal contents as well as of viable bacteria translocating from the intestines to extra-intestinal compartments including MLN, liver, lung and spleen, respective intestinal samples and *ex vivo* biopsies were homogenized in sterile phosphate buffered saline (PBS, Gibco life technologies, UK) and analyzed in serial dilutions on respective solid media as described earlier [9, 11, 20]. Cardiac blood was directly streaked onto solid media. Bacteria were grown at 37°C for at least two days under aerobic, microaerobic and anaerobic conditions.

### Molecular analysis of the human donor suspension and the ileal microbiota

DNA was extracted from the human donor suspension and intestinal luminal content as described previously [9]. In brief, DNA was quantified by using Quant-iT PicoGreen reagent (Invitrogen, UK) and adjusted to 1 ng per μL. Then, main bacterial groups abundant in the murine and human intestinal microbiota were assessed by quantitative real-time polymerase chain reaction (qRT-PCR) with species-, genera- or group-specific 16S rRNA gene primers (Tib MolBiol, Germany) and numbers of 16S rRNA gene copies per ng DNA of each sample determined as described previously [14, 15, 21, 22].

### Cytokine detection

*Ex vivo* biopsies of approximately 1cm^2^ (ileum and colon cut longitudinally) were washed in PBS and placed in 24-flat-bottom well culture plates (Falcon, Germany) containing 500 mL serum-free RPMI 1640 medium (Gibco, life technologies) supplemented with penicillin (100 U/mL, Biochrom, Germany) and streptomycin (100 μg/mL; Biochrom). After 18 hours at 37°C, culture supernatants and serum samples were tested for IFN-γ, TNF, MCP, IL-12p70, IL-6 and IL-10 by the Mouse Inflammation Cytometric Bead Assay (CBA; BD Bioscience) on a BD FACSCanto II flow cytometer (BD Bioscience). Nitric oxide (NO) was measured by the Griess reaction as described earlier [9].

### Statistical analysis

Mean values, medians, standard deviations (SD) and levels of significance were determined using appropriate tests as indicated (two-tailed Student´s t-test, Mann-Whitney U test). Two-sided probability (*p*) values ≤0.05 were considered significant. Experiments were repeated at least twice.

## Results

### Generation of human microbiota associated mice by fecal microbiota transplantation

**Fig 1:**
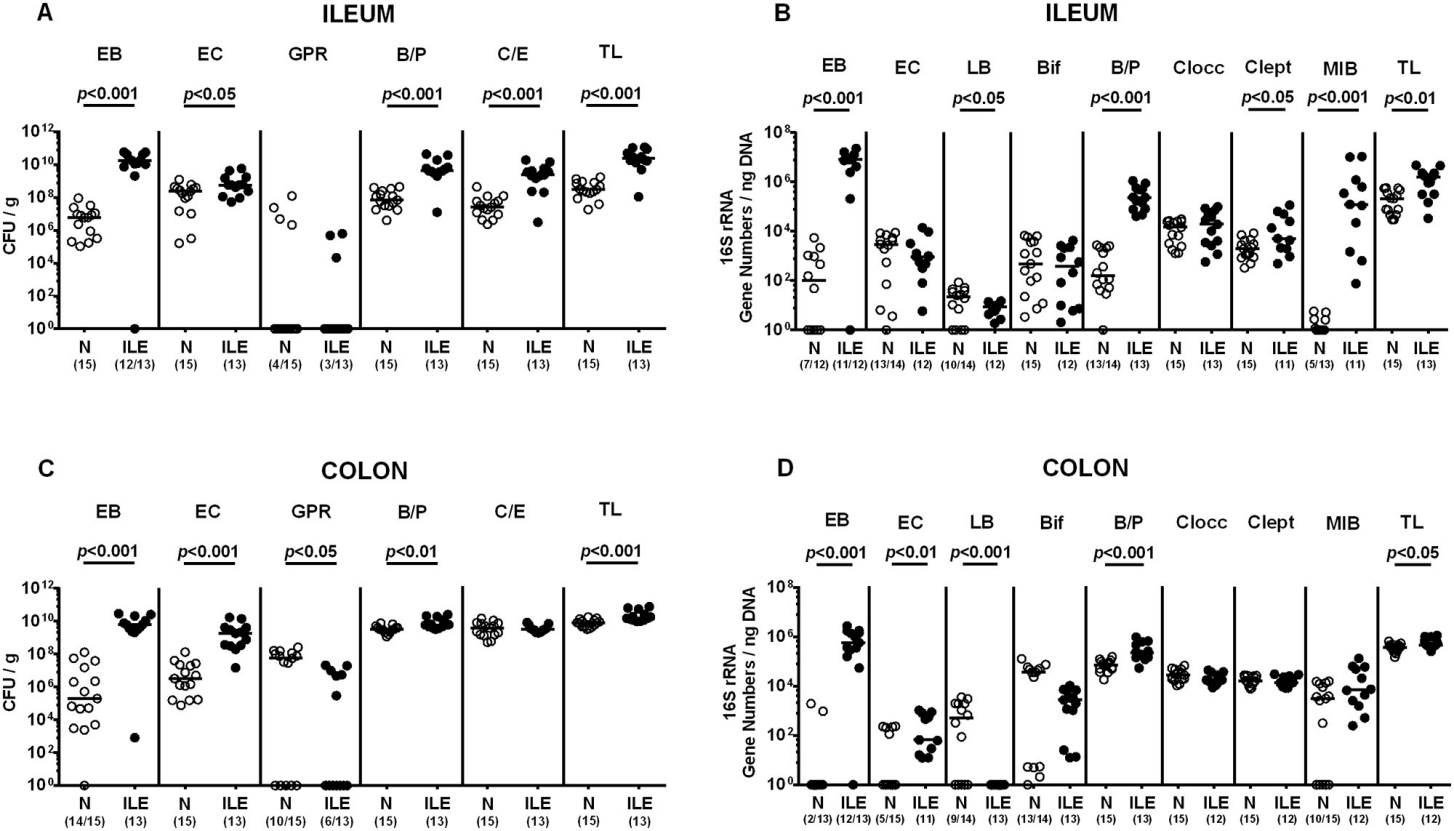
Intestinal microbiota composition in human microbiota associated mice suffering from acute ileitis. Human microbiota associated (hma) were perorally infected with *T*. *gondii* strain ME49 to induce acute ileitis as described in methods. Main intestinal bacterial groups abundant in the ileum (**A, B**) and the colon lumen (**C, D**) of hma mice were quantitatively assessed applying both culture (**A, C**) and culture-independent (i.e. molecular 16S rRNA based; **B, D**) methods 7 days following ileitis induction (ILE, filled circles). Noninfected hma mice served as controls (N, open circles). Numbers of enterobacteria (EB), enterococci (EC), Gram-positive rods (GPR), *Bacteroides / Prevotella* spp. (B/P), *Clostridium / Eubacterium* spp. (C/E) and the total bacterial loads (TL) are expressed as colony forming units per gram feces (CFU / g). 16S rRNA of the main intestinal bacterial groups including enterobacteria (EB), enterococci (EC), lactobacilli (LB), bifidobacteria (Bif), *Bacteroides / Prevotella* species (B/P), *Clostridium coccoides* group (Clocc), *Clostridium leptum* group (Clept), *Mouse Intestinal Bacteroides* (MIB) and the total eubacterial loads (TL) are expressed as gene numbers per ng DNA. Numbers of animals harboring the respective bacterial group out of the total number of analyzed mice are given in parentheses. Medians (black bars) and significance levels (*p*-values) determined by Student’s t test and Mann-Whitney U test are indicated. Data shown are pooled from three independent experiments.

In the present study we investigated intestinal, extra-intestinal and systemic sequelae of acute *T*. *gondii* induced ileitis in (with respect to the intestinal microbiota composition) “humanized” mice. To assure stable intestinal colonization of mice with a complex human microbiota, we initially generated secondary abiotic animals by quintuple antibiotic treatment for eight weeks. Subsequently, mice with a virtually depleted intestinal microbiota were subjected to peroral transplantation of human microbiota generated from five healthy human individuals on three consecutive days. Quantitative cultural as well as culture-independent (i.e. 16S rRNA based molecular) analyses revealed comparable microbiota compositions of respective human fecal suspensions (S1 Fig). Within three weeks the human microbiota had stably established within the murine intestinal tract (Fig 1).

### Microbiota shifts during acute ileitis of human microbiota associated mice

In order to induce ileitis, hma mice were perorally infected with a high dose (i.e. 50 cysts) of *T*. *gondii* strain ME49 on day 0. As assessed by culture, the ileal loads of the main intestinal commensals including aerobic enterobacteria and enterococci as well as the obligate anaerobic *Bacteroides / Prevotella* spp. and *Clostridium / Eubacterium* spp. increased in hma mice within seven days post ileitis induction (*p*<0.05–0.001; Fig 1A). Molecular analyses additionally assessing fastidious or even uncultivable commensals confirmed cultural results and further revealed increased gene numbers of *Mouse Intestinal Bacteroides* (*p*<0.001), *Clostridium leptum* (*p*<0.05), but not *C*. *coccoides* (n.s.), whereas decreased 16S rRNA copies of lactobacilli (*p*<0.05) could be determined at day 7 p.i. as compared to uninfected hma control mice (Fig 1B). Notably, luminal microbiota shifts were not restricted to the inflamed distal small intestines as indicated by distinctly elevated enterobacteria, enterococci and *Bacteroides / Prevotella* spp. in the colonic lumen of *T*. *gondii* infected hma mice (*p*<0.01–0.001; Fig 1C and 1D). Conversely, numbers of cultivable Gram-positive rods (such as *Bacillus* spp. and lactobacilli; p<0.05; Fig 1C) as well as lactobacilli 16S rRNA gene copies decreased in the colon of hma mice during ileitis development (p<0.001; Fig 1D). Hence, acute ileitis is accompanied by pronounced shifts in the intestinal microbiota composition of hma mice.

### Clinical, macroscopic and microscopic sequelae in *T*. *gondii* infected human microbiota associated mice

**Fig 2:**
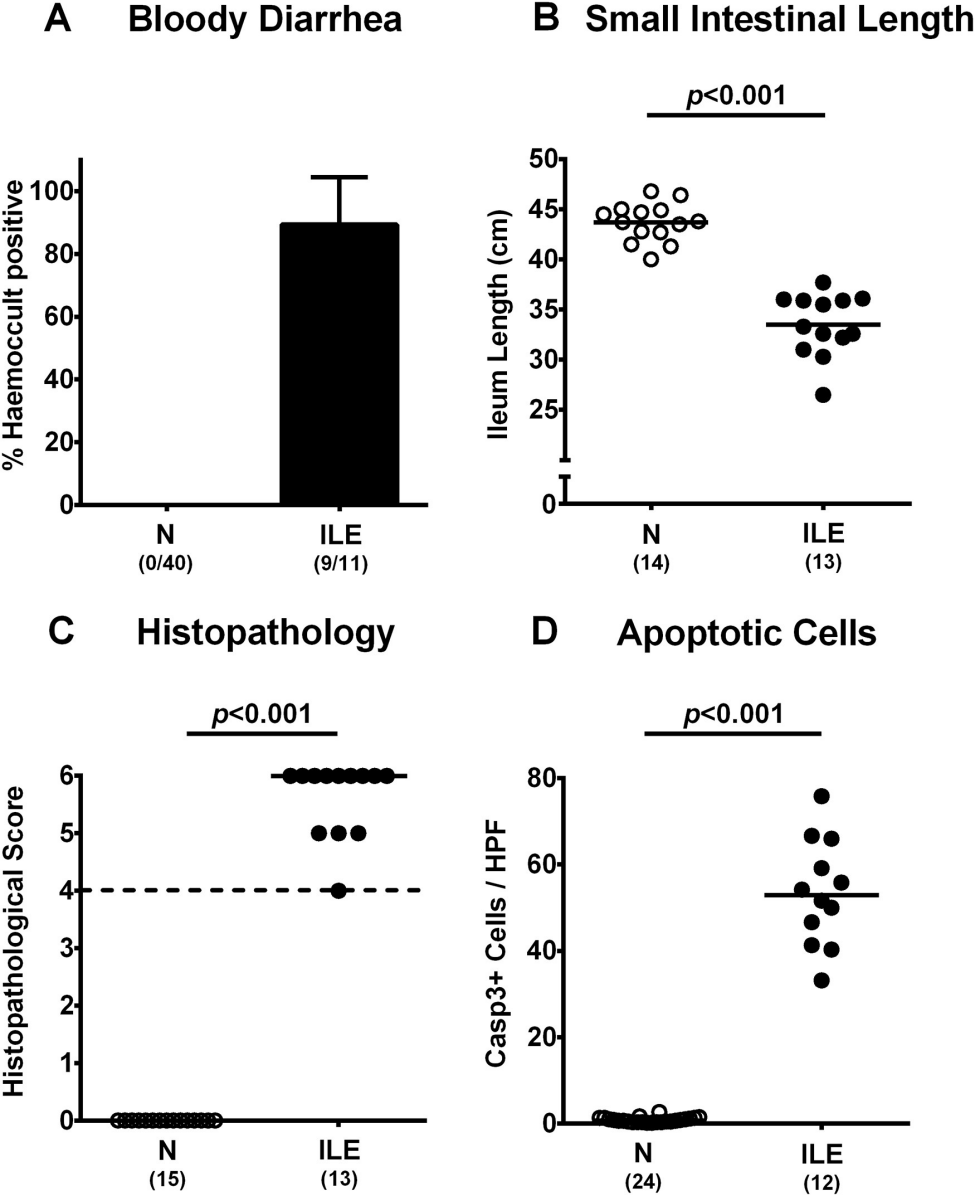
Clinical, macroscopic and microscopic sequelae in human microbiota associated mice suffering from acute ileitis. Human microbiota associated (hma) mice were perorally infected with *T*. *gondii* strain ME49 to induce acute ileitis (ILE; filled symbols). Noninfected hma mice served as controls (N, open symbols). Clinical, macroscopic and microscopic intestinal changes were assessed at day 7 following ileitis induction: **(A)** Abundance of blood was determined in fecal samples by the Guajac (Haemoccult) method. Means, standard deviations and numbers of fecal blood positive mice out of the total numbers of analyzed animals are given in parentheses. **(B)** Absolute small intestinal lengths were measured (in cm) and **(C)** histopathological changes were determined in H&E stained ileal paraffin sections applying a standardized scoring system (see methods). Scores ≥4 (dotted line) indicate severe inflammation with necrosis. **(D)** The average numbers of apoptotic cells (positive for caspase 3; Casp3+) from at least six high-power fields (HPF, 400 x magnification) per animal were determined microscopically in immunohistochemically stained ileal paraffin sections. Numbers of animals (in parentheses), medians and significance levels (*p*-values) determined by Students-t test and Mann-Whitney U test are indicated. Data shown were pooled from three independent experiments.

Until day 7 following *T*. *gondii* infection hma were clinically severely compromised and exhibited wasting and bloody diarrhea in almost 90% of cases (Fig 2A). Since intestinal inflammation is known to be associated with significant shortening of the inflamed gut in conventional mice [9, 14, 23, 24], we measured small intestinal lengths of hma mice at necropsy. In fact, *T*. *gondii* infected hma mice displayed shorter small intestines as compared to noninfected hma control animals (*p*<0.001, Fig 2B). Macroscopic sequelae of *T*. *gondii* infection were supported by microscopic intestinal inflammatory changes. Seven days post ileitis induction, all hma mice displayed high histopathological scores indicating severe ileal inflammation with destruction of the villous architecture, cellular shedding into the lumen and massive necrosis (*p*<0.001 vs noninfected controls; Fig 2C, S2A Fig). Furthermore, ileal apoptotic cell numbers increased multifold upon ileitis induction (*p*<0.001; Fig 2D, S2B Fig). Hence, *T*. *gondii* infection of hma mice results in severe macroscopic as well as microscopic inflammatory sequelae.

### Intestinal inflammatory immune responses upon *T*. *gondii* infection of human microbiota associated mice

**Fig 3:**
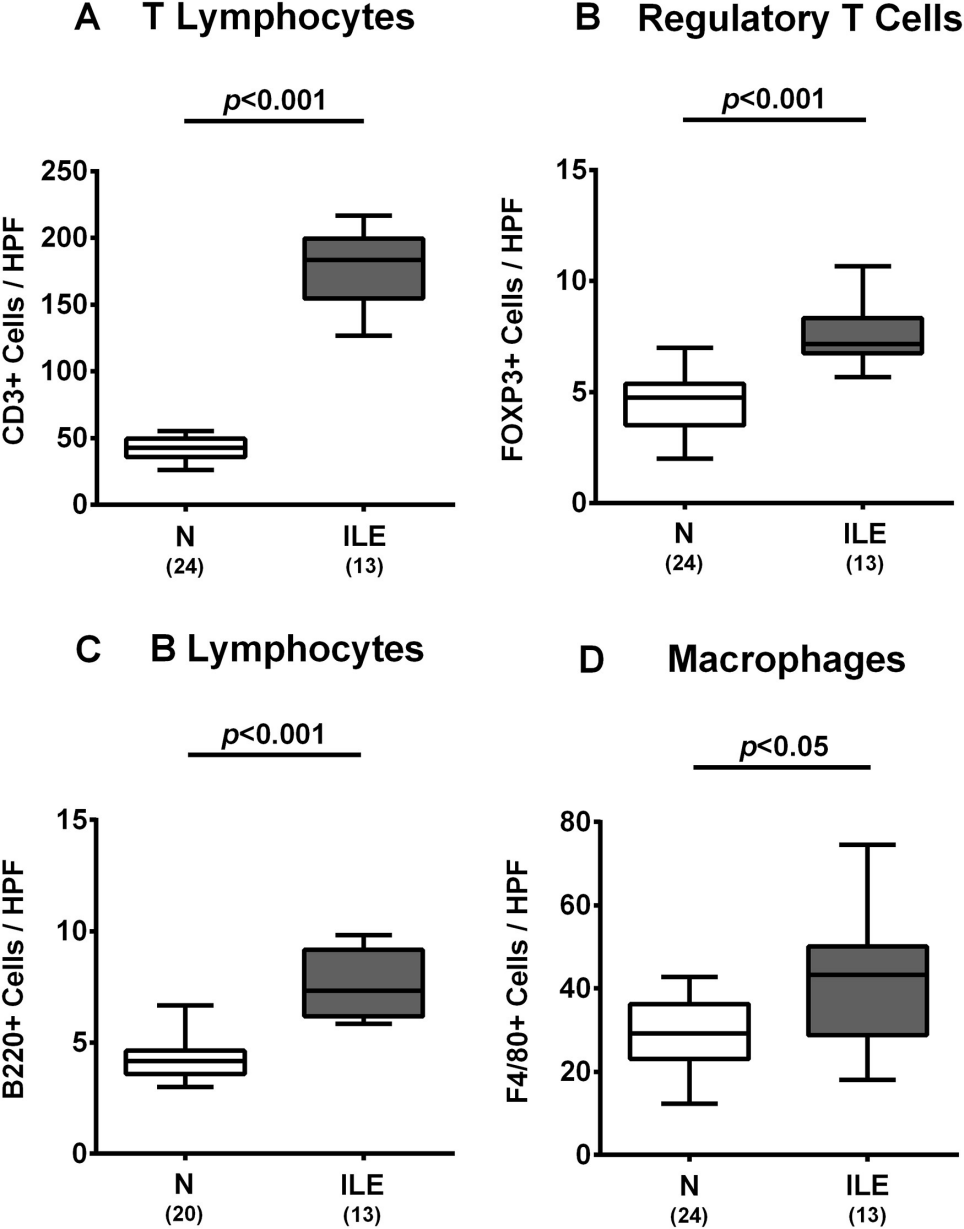
Small intestinal immune cell responses in human microbiota associated mice suffering from acute ileitis. Human microbiota associated (hma) mice were perorally infected with *T*. *gondii* strain ME49 to induce acute ileitis (ILE; grey boxes). Noninfected hma mice served as controls (N, white boxes). The average numbers of ileal **(A)** T lymphocytes (positive for CD3), **(B)** regulatory T cells (positive for FOXP3), **(C)** B lymphocytes (positive for B220), and **(D)** macrophages (positive for F4/80) from six high power fields (HPF, 400 x magnification) per animal were determined microscopically in immunohistochemically stained ileal paraffin sections at day 7 post ileitis induction. Box plots represent the 75th % and 25th % percentiles of the medians (black bar inside the boxes). Total range and significance levels (*p*-values) determined by the Student’s t test and Mann-Whitney U test and numbers of mice (in parentheses) are indicated. Data shown were pooled from three independent experiments.

**Fig 4:**
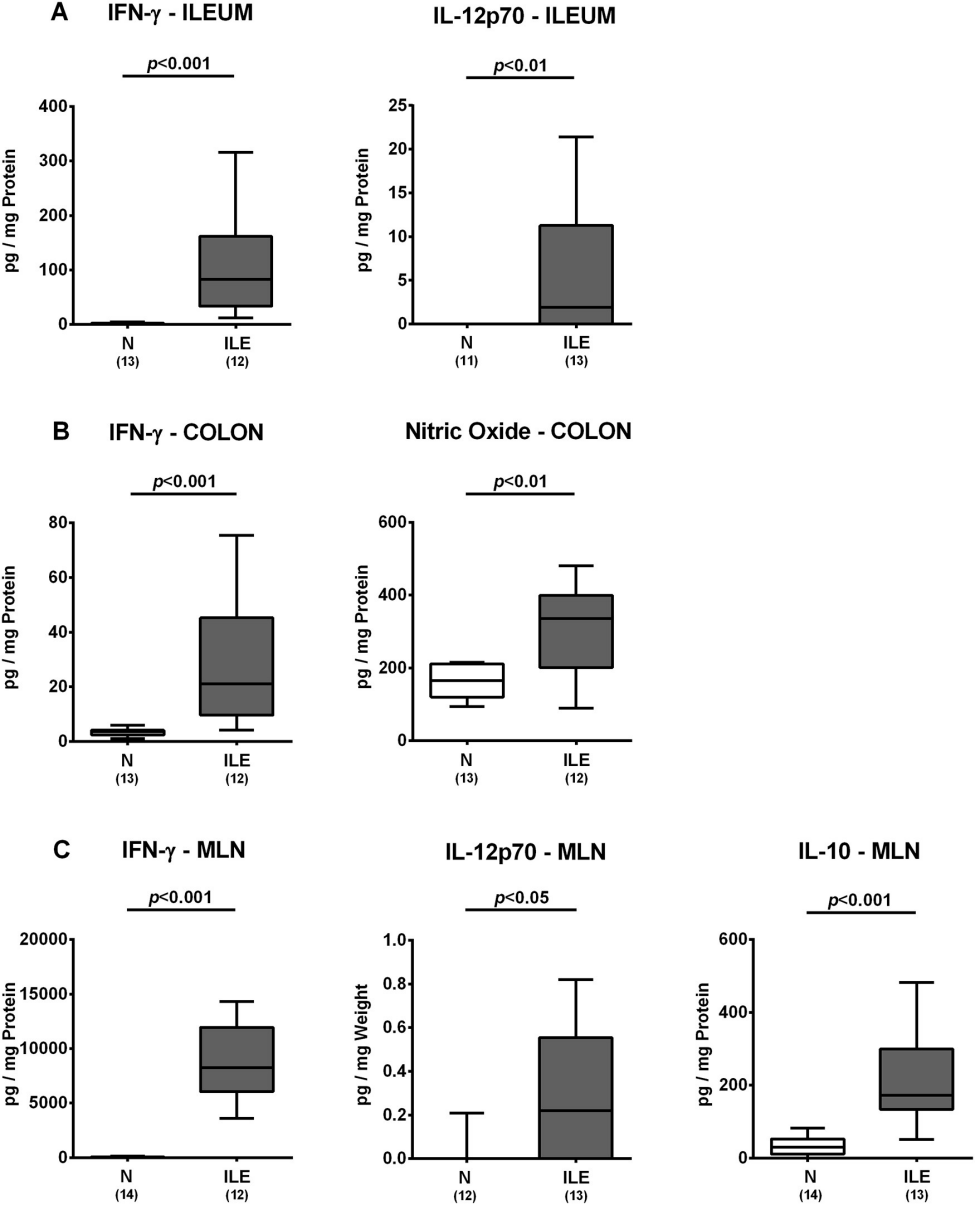
Intestinal cytokine responses in human microbiota associated mice suffering from acute ileitis. Human microbiota associated (hma) mice were perorally infected with *T*. *gondii* strain ME49 to induce acute ileitis (ILE; grey boxes). Noninfected hma mice served as controls (N, white boxes). At day 7 post ileitis induction secretion of distinct pro- and anti-inflammatory cytokines (as indicated) were determined in *ex vivo* biopsies derived from distinct intestinal compartments including **(A)** ileum **(B)** colon and **(C)** mesenteric lymph nodes (MLN). Box plots represent the 75th % and 25th % percentiles of the medians (black bar inside the boxes). Total range and significance levels (*p*-values) determined by the Student’s t test and Mann-Whitney U test and numbers of mice (in parentheses) are indicated. Data shown were pooled from three independent experiments.

We further surveyed microscopic inflammatory sequelae and therefore quantitatively assessed small intestinal immune cell responses in hma mice at day 7 post ileitis induction. Numbers of T and B lymphocytes, Treg as well as of macrophages and monocytes substantially increased within the ileal mucosa and lamina propria of hma mice until day 7 p.i. (*p*<0.05–0.001; Fig 3, S2C–S2F Fig). Ileal increases in innate and adaptive immune cell populations were accompanied by elevated small intestinal secretion of pro-inflammatory cytokines such as IFN-γ and IL-12p70 at day 7 p.i. (*p*<0.001 and *p*<0.01, respectively; Fig 4A). Remarkably, pro-inflammatory responses were not restricted to the ileum, given that *T*. *gondii* infection also resulted in increased IFN-γ and nitric oxide concentrations in colonic *ex vivo* biopsies (*p*<0.001 and *p*<0.01, respectively; Fig 4B), whereas IFN-γ and IL-12p70 levels increased multifold in MLN of hma mice with acute ileitis (*p*<0.001 and *p*<0.05, respectively; Fig 4C). Furthermore, concentrations of the anti-inflammatory cytokine IL-10 were higher in MLN of *T*. *gondii* infected as compared to noninfected hma mice (*p*<0.001; Fig 4). Hence, peroral *T*. *gondii* infection induces marked pro-inflammatory immune responses in the intestines of hma mice.

### Bacterial translocation in *T*. *gondii* infected human microbiota associated mice

**Fig 5:**
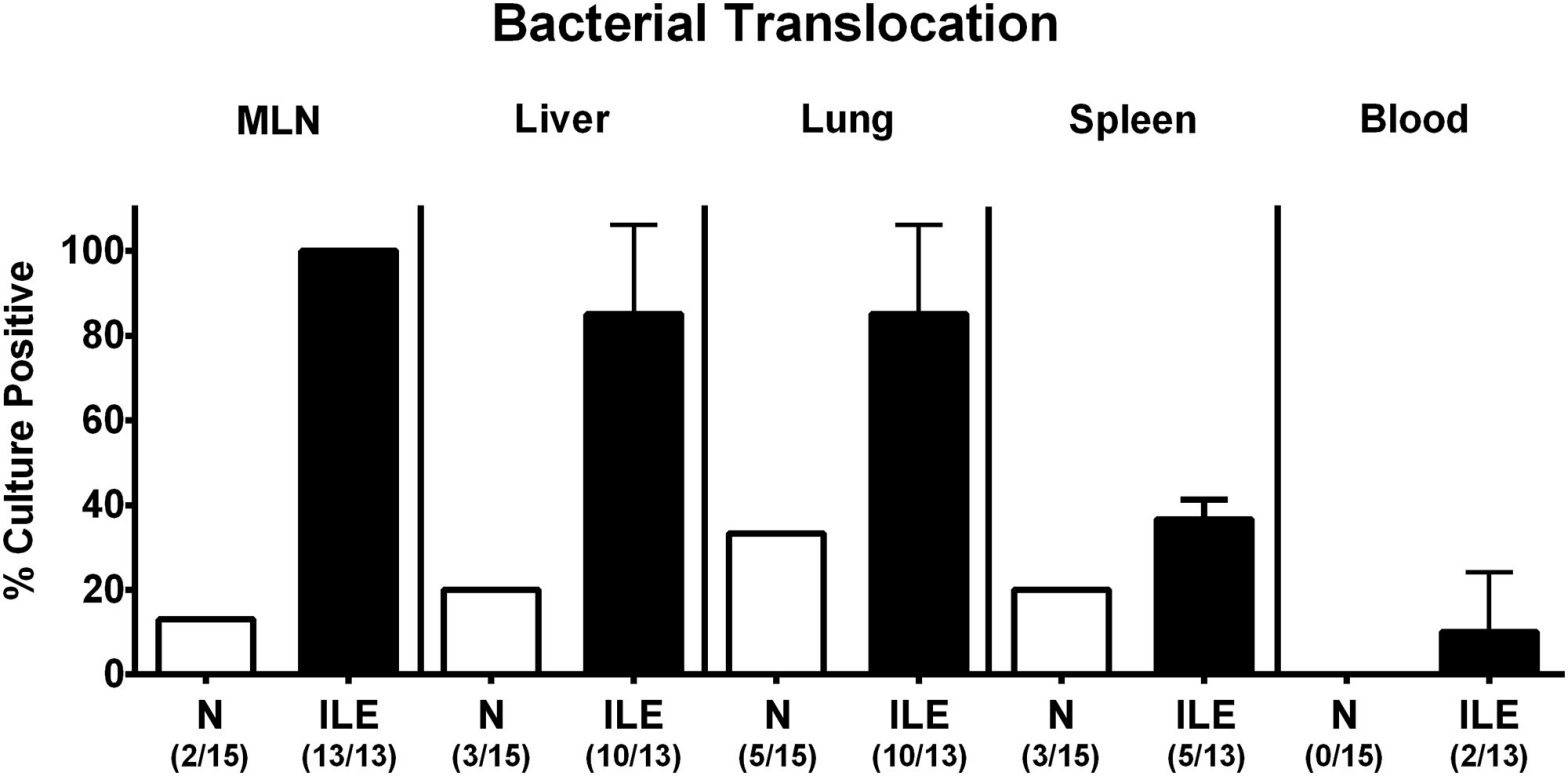
Translocating intestinal bacteria in human microbiota associated mice suffering from acute ileitis. Human microbiota associated (Hma) mice were perorally infected with *T*. *gondii* strain ME49 to induce acute ileitis (ILE, filled bars). Noninfected hma mice served as controls (N, open bars). At day 7 post ileitis induction rates of viable intestinal bacterial species translocating to extra-intestinal and systemic compartments were determined by cultivation of *ex vivo* biopsies derived from mesenteric lymph nodes (MLN), liver, lung and spleen and of cardiac blood. Mean translocation rates (in %) ± standard deviations and numbers of culture-positive samples out of the total number of analyzed animals are indicated in parentheses. Data shown were pooled from three independent experiments.

**Fig 6:**
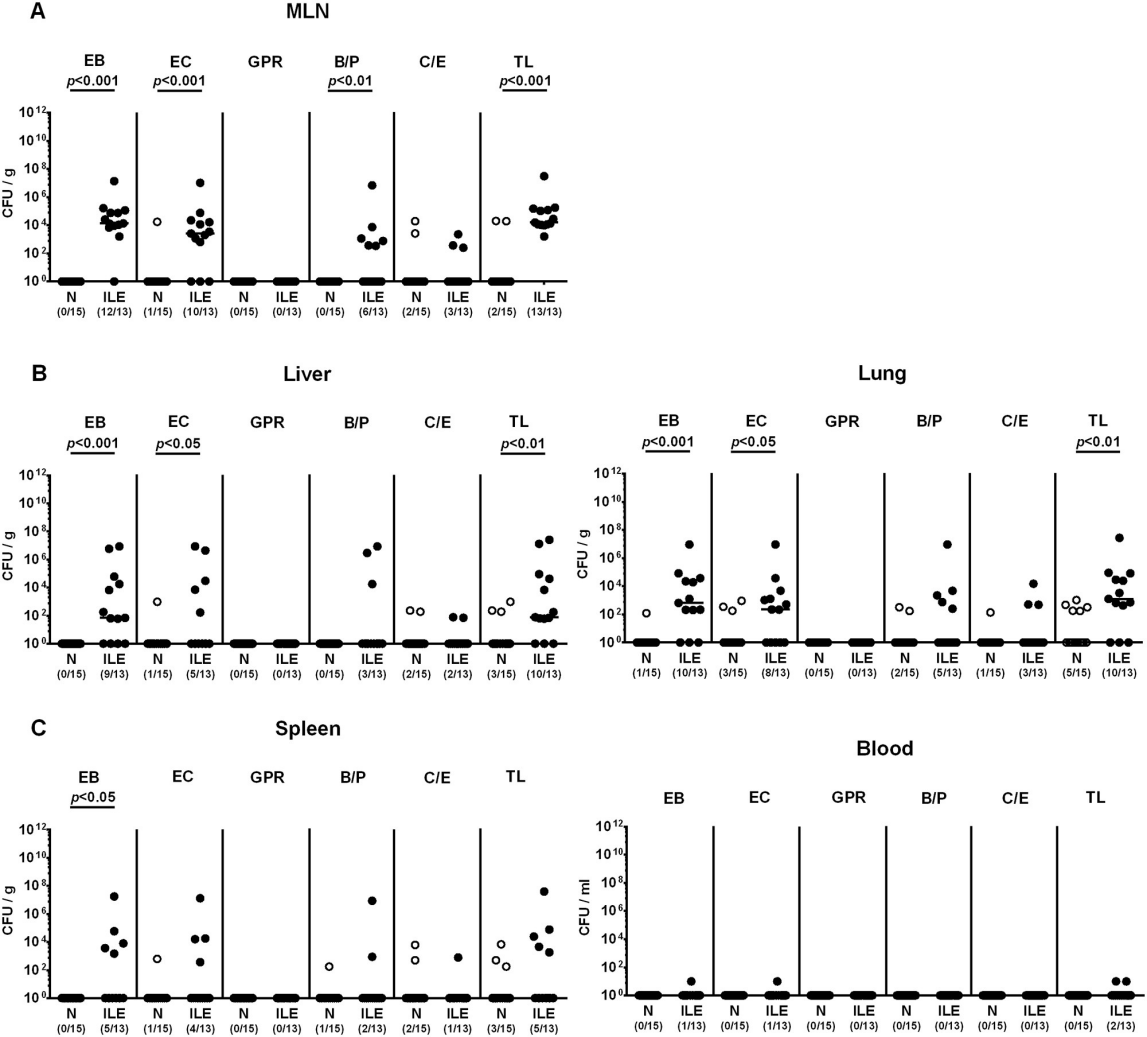
Intestinal bacteria translocating to extra-intestinal compartments in human microbiota associated mice suffering from acute ileitis. Human microbiota associated (hma) mice were perorally infected with *T*. *gondii* strain ME49 to induce acute ileitis (ILE, filled circles). Noninfected hma mice served as controls (N, open circles). At day 7 post ileitis induction viable intestinal bacteria translocating to **(A)** MLN, **(B)** extra-intestinal (i.e. liver and lung) and **(C)** systemic compartments (i.e. spleen and blood) were quantitatively determined by culture of respective *ex vivo* biopsies and cardiac blood. Numbers of enterobacteria (EB), enterococci (EC), Gram-positive rods (GPR), *Bacteroides / Prevotella* species (B/P), *Clostridium / Eubacterium* species (C/E) and the total bacterial loads (TL) were expressed as colony forming units per gram organ homogenate or ml blood (CFU/g; CFU/ml). Numbers of culture-positive samples out of the total number of analyzed mice are given in parentheses. Medians (black bars) and significance levels (*p*-values) determined by Mann-Whitney U test are indicated. Data shown were pooled from three independent experiments.

We next addressed whether viable intestinal commensal bacteria had translocated from the inflamed (and hence leaky) ilea to extra-intestinal including systemic compartments. As assessed by direct plating, commensals such as enterobacteria, enterococci and *Bacteroides / Prevotella* spp. that had overgrown the inflamed ileal lumen during ileitis could be isolated from MLN of all mice and from liver and lung in 85.0 ± 21.2% of cases at day 7 p.i. (Figs 5 and 6). Remarkably, viable bacteria could also be detected in systemic compartments as indicated by translocation rates of 36.7 ±4.7% and 10.0 ±14.1% in spleen and cardiac blood, respectively (Figs 5 and 6). Of note, commensal bacteria could be cultured from respective organ homogenates and blood derived from naive mice in single cases only (Figs 5 and 6).

### Extra-intestinal including systemic inflammatory responses in *T*. *gondii* infected human microbiota associated mice

**Fig 7:**
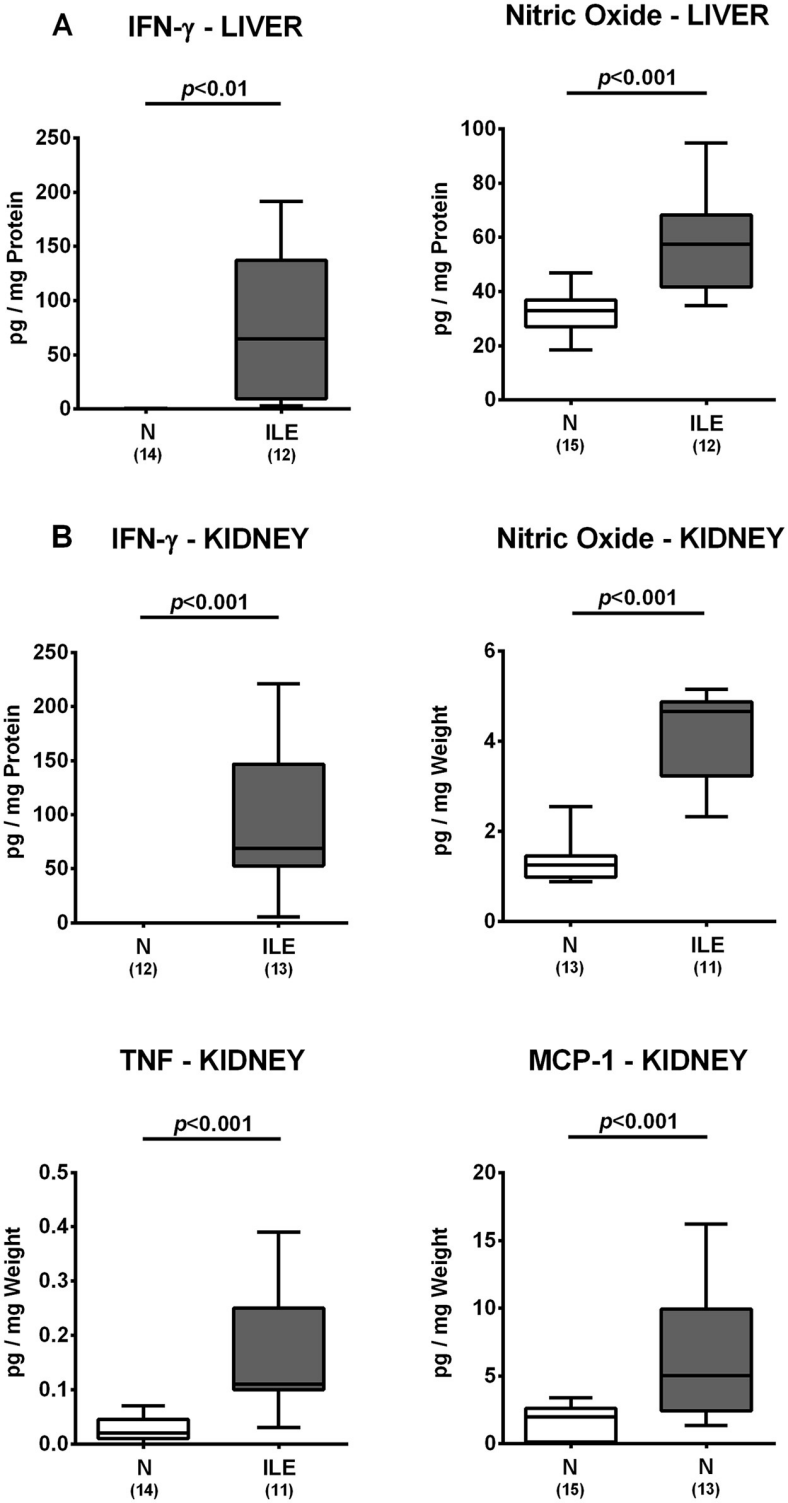
Extra-intestinal cytokine responses in human microbiota associated mice suffering from acute ileitis. Human microbiota associated (hma) mice were perorally infected with *T*. *gondii* strain ME49 to induce acute ileitis (ILE; grey boxes). Noninfected hma mice served as controls (N, white boxes). At day 7 post ileitis induction secretion of distinct pro-inflammatory cytokines (as indicated) were determined in *ex vivo* biopsies derived from distinct extra-intestinal compartments including **(A)** liver and **(B)** kidney. Box plots represent the 75th % and 25th % percentiles of the medians (black bar inside the boxes). Total range and significance levels (*p*-values) determined by the Student’s t test and Mann-Whitney U test and numbers of mice (in parentheses) are indicated. Data shown were pooled from three independent experiments.

**Fig 8:**
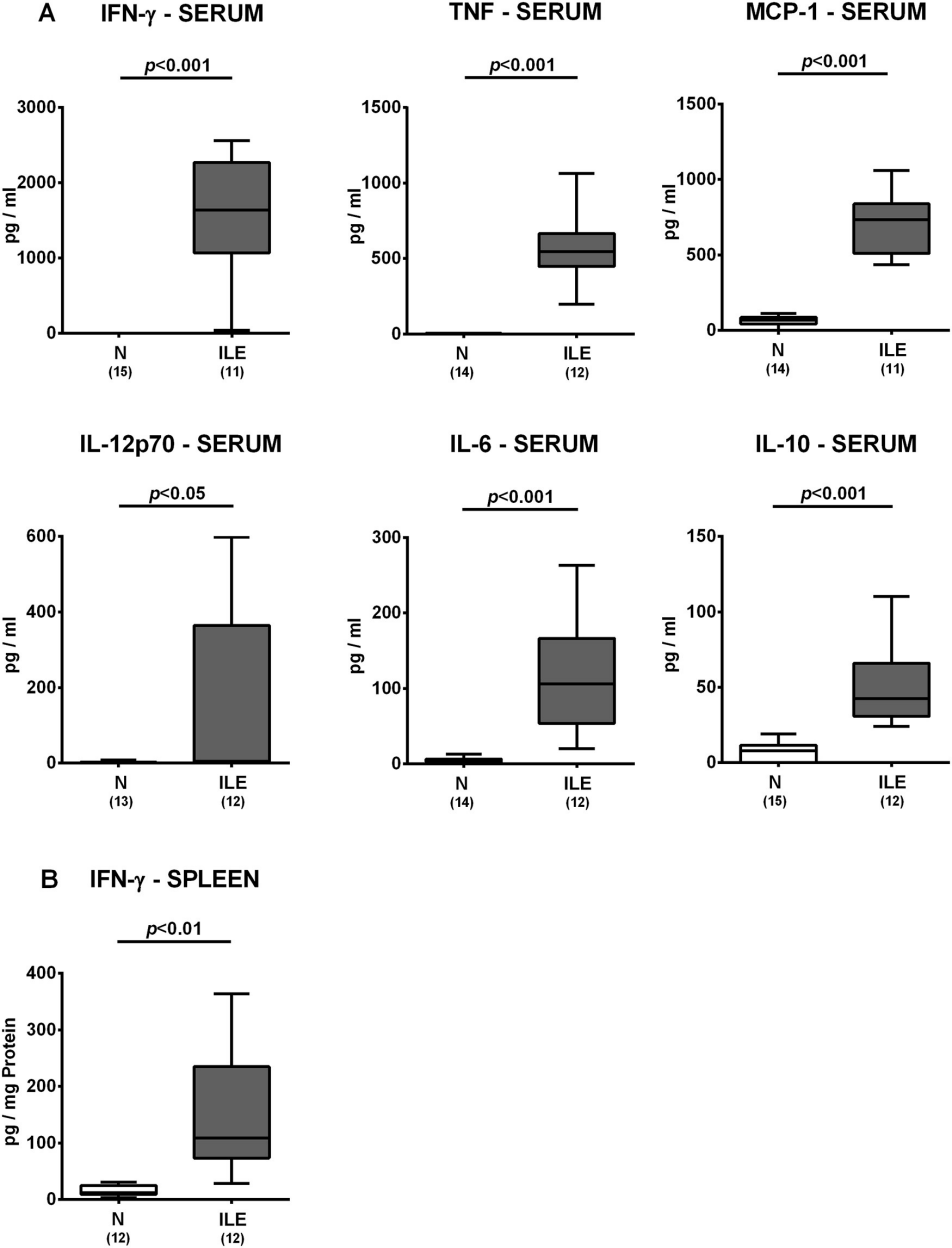
Systemic cytokine responses in human microbiota associated mice suffering from acute ileitis. Human microbiota associated mice were perorally infected with *T*. *gondii* strain ME49 to induce acute ileitis (ILE; grey boxes). Noninfected hma mice served as controls (N, white boxes). At day 7 following ileitis induction secretion of distinct pro- and anti-inflammatory cytokines were determined in systemic compartments including **(A)** serum and **(B)** spleen (as indicated). Box plots represent the 75th % and 25th % percentiles of the medians (black bar inside the boxes). Total range and significance levels (*p*-values) determined by the Student’s t test and Mann-Whitney U test and numbers of mice (in parentheses) are indicated. Data shown were pooled from three independent experiments.

We next assessed whether *T*. *gondii* induced inflammatory responses could also be observed in extra-intestinal compartments. In fact, IFN-γ and nitric oxide concentrations were multifold higher in *ex vivo* biopsies derived from liver and kidney of hma mice at day 7 p.i. as compared to noninfected hma control animals (*p*<0.01–0.001; Fig 7). In addition, renal TNF and MCP-1 levels increased in hma mice upon ileitis induction (*p*<0.001; Fig 7B). Remarkably, *T*. *gondii* infection of hma mice also resulted in pronounced systemic pro-inflammatory immune responses, given that serum levels of pro-inflammatory cytokines including IFN-γ, TNF, MCP-1, IL-12p70 and IL-6 were all multifold elevated at day 7 p.i. (*p*<0.05–0.001 vs noninfected controls; Fig 8A) and paralleled by increased IFN-γ secretion in splenic *ex vivo* biopsies taken from hma mice with ileitis (*p*<0.01 vs noninfected controls; Fig 8B). Furthermore, serum concentrations of the anti-inflammatory cytokine IL-10 were higher in infected as compared to naive hma mice (*p*<0.001; Fig 8A). Hence, *T*. *gondii* infection of hma mice was accompanied by translocation of commensal bacteria from the inflamed small intestines to extra-intestinal compartments and resulted not only in intestinal, but also extra-intestinal and systemic inflammatory immune responses.

## Discussion

In the present study we surveyed the suitability of (with respect to the microbiota) “humanized” mice to study the interactions between pathogen, human commensal microbiota and host immunity during acute *T*. *gondii* induced ileitis. Therefore, secondary abiotic mice were generated by broad-spectrum antibiotic treatment and subsequently subjected to peroral FMT. Despite originating from a foreign host, the complex human microbiota was able to fully establish in the murine intestinal tract within three weeks before ileitis induction. In addition to a “classical” culture-based survey of the intestinal microbiota composition we applied culture-independent (i.e. quantitative 16S rRNA based real-time PCR) methods to also assess fastidious or even uncultivable bacteria including bifidobacteria, clostridia and *Mouse Intestinal Bacteroides*. One needs to take into consideration, however, that it can not be distinguished whether amplified 16S rRNA gene copies were derived form viable or “dead” bacterial cells. The here presented comprehensive survey of the intestinal microbiota in “humanized” mice was further supported by our previous studies where the human microbiota composition was stable in the murine gastrointestinal tract for at least six weeks following FMT [14, 25]. In addition, it could be shown that the microbiota of hma mice remained stable over several generations [26]. High dose infection of hma mice with at least 50 cysts of *T*. *gondii* resulted in severe macroscopic and microscopic pro-inflammatory sequelae as indicated by wasting, bloody diarrhea and acute transmural ileitis with both apoptosis and necrosis that were comparable to disease outcomes in *T*. *gondii* infected conventionally colonized mice as shown in several previous reports [5, 9, 11, 17, 23, 24, 27–31]. In both conventional and hma mice this non-selflimiting T cell-driven inflammatory scenario was further characterized by excessive ileal secretion of pro-inflammatory cytokines including IFN-γ and IL-12. So far, the ileum has been described as the exclusive intestinal predilection site of high dose *T*. *gondii* infection [3]. Strikingly, we were able to show here that *T*. *gondii* infection resulted in enhanced secretion of pro-inflammatory mediators (including IFN-γ and nitric oxide) also in the large intestines.

The intriguing role of the intestinal microbiota in initiation and perpetuation of *T*. *gondii* induced ileitis could be proven by our previous and actual *in vivo* studies, given that secondary abiotic mice were virtually unaffected from *T*. *gondii* infection, whereas with a complex microbiota recolonized mice (irrespective whether of murine [9] or human origin as shown here) were exhibiting the full-blown disease. Similar to conventional mice [9, 11], small intestinal inflammation was accompanied by substantial shifts in the ileal microbiota composition of hma mice towards a marked overgrowth of the inflamed ileal, but also (to a lesser extent) colonic lumen with Gram-negative commensal species such as *E*. *coli* and *Bacteroides / Prevotella* spp. Importantly, comparable microbiota shifts can also be observed in Crohn’s disease patients during the acute stage [32–35]. Similar to conventionally colonized mice, the in the course of the inflammatory process compromised intestinal epithelial barrier function (i.e. “leaky gut”) resulted in translocation of viable intestinal commensals overgrowing the ileal lumen to extra-intestinal including systemic compartments (i.e. liver, lung, spleen and blood), further aggravating the inflammatory process as indicated by an exaggerated increase in inflammatory cytokines (including IFN-, TNF, MCP-1, IL-12, IL-6 and IL-10) in the serum taken from hma mice with acute ileitis (“cytokine storm” [5]). Hence, our results further emphasize the feasibility of the hma mouse model in investigating microbiota-host interactions and their underlying molecular mechanisms during intestinal inflammation mimicking human flora conditions.

The hma mouse model has been further proven valuable in dissecting the interplay between enteropathogens such as *Campylobacter jejuni*, the host microbiota and immunity [8, 13, 14]. Whereas conventionally colonized mice could not be infected by the pathogen, hma mice harbored *C*. *jejuni* in their gastrointestinal tract at high loads and exhibited pro-inflammatory key features of human campylobacteriosis [14, 36]. Very recently, Collins and colleagues presented an hma mouse model of recurrent *Clostridium difficile* infection during antimicrobial therapy [26]. Hma rodents have been further used in intestinal inflammation models in order to investigate host susceptibility to disease depending on a defined human microbiota that had been derived from healthy and diseased individuals [37, 38]. Furthermore, hma isolator-raised (formerly germ-free) mice were applied in several studies for dissecting intestinal microbiota changes upon different diets and during obesity [39–42]. Since immunological differentiation and stimulation are highly microbiota dependent, secondary abiotic mice have the advantage of a fully developed immune system [8]. This point of view is shared by previous studies reporting pivotal differences in phenotypes already under basal conditions and even more pronounced in various disease states when applying isolator-raised germfree and conventionally reared mice [43–46].

We conclude that hma secondary abiotic mice that were generated by antibiotic treatment of conventionally reared mice represent valuable tools for studies investigating the molecular mechanism underlying distinct microbiota—host interactions in health and disease including acute intestinal inflammation.

## Supporting information

S1 Fig Microbiota composition of human donor feces. Before fecal microbiota transplantation of secondary abiotic mice, main intestinal bacterial groups were quantitatively assessed in human donor fecal suspensions. **(A)** Numbers of viable enterobacteria (EB), enterococci (EC), Gram-positive rods (GPR), *Bacteroides / Prevotella* species (B/P), *Clostridium / Eubacterium* species (C/E) and the total bacterial load (TL) were determined by culture and expressed as colony forming units (CFU) per ml suspension. **(B)** 16S rRNA of the main intestinal bacterial commensals including enterobacteria (EB), enterococci (EC), lactobacilli (LB), bifidobacteria (Bif), *Bacteroides / Prevotella* species (B/P), *Clostridium coccoides* group (Clocc), *Clostridium leptum* group (Clept), *Mouse Intestinal Bacteroides* (MIB) and the total eubacterial load (TL) were analyzed by quantitative RT-PCR and expressed as gene numbers per ng DNA. Data shown are representative for at least three independent experiments.
(TIFF)

S2 Fig Immunohistopathological changes in small intestines of human microbiota associated mice suffering from acute ileitis. Human microbiota associated (hma) and conventionally colonized (SPF) mice were perorally infected with *T*. *gondii* strain ME49 to induce acute ileitis. Noninfected mice served as respective naive controls. Small intestinal immunohistopathological changes were assessed at day (d) 7 following ileitis induction in ileal paraffin sections stained with **(A)** hematoxylin & eosin or antibodies against **(B)** caspase-3, **(C)** CD3, **(D)** FOXP3, **(E)** B220 or **(F)** F4/80. Representative photomicrographs from three independent experiments are shown (A, C, D: 100 x magnification, scale bars 100 μm; B, E, F: 400 x magnification, scale bars 20 μm).
(PDF)
