# Supplementary figures and images for: Intestinal, extra-intestinal and systemic sequelae of Toxoplasma gondii induced acute ileitis in mice harboring a human gut microbiota
Source: PLoS One. 2017 Apr 17;12(4):e0176144. doi: 10.1371/journal.pone.0176144 (PMC5393883; doi:10.1371/journal.pone.0176144)

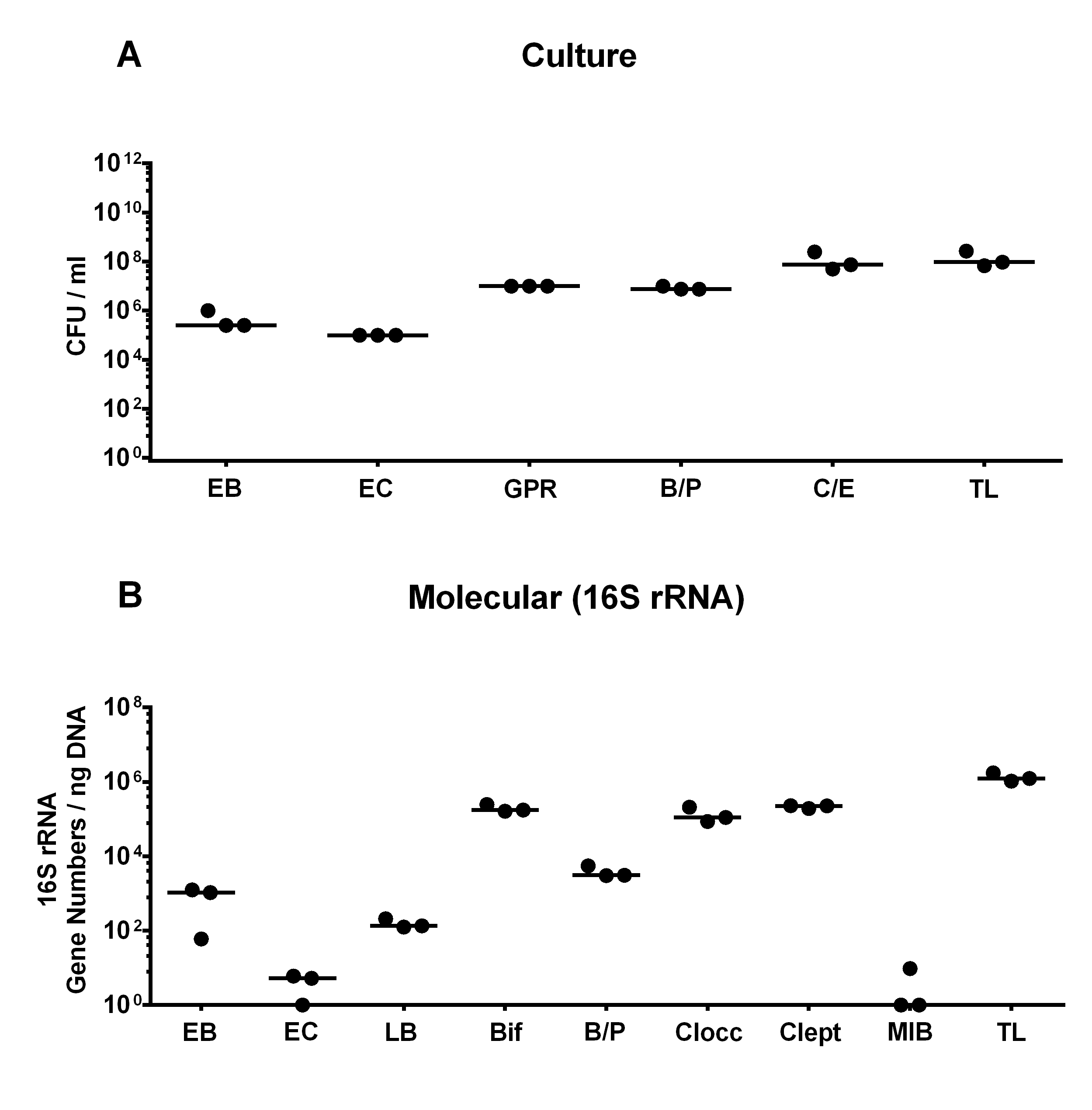

Supplement: S1 Fig — Before fecal microbiota transplantation of secondary abiotic mice, main intestinal bacterial groups were quantitatively assessed in human donor fecal suspensions. (A) Numbers of viable enterobacteria (EB), enterococci (EC), Gram-positive rods (GPR), Bacteroides / Prevotella species (B/P), Clostridium / Eubacterium species (C/E) and the total bacterial load (TL) were determined by culture and expressed as colony forming units (CFU) per ml suspension. (B) 16S rRNA of the main intestinal bacterial commensals including enterobacteria (EB), enterococci (EC), lactobacilli (LB), bifidobacteria (Bif), Bacteroides / Prevotella species (B/P), Clostridium coccoides group (Clocc), Clostridium leptum group (Clept), Mouse Intestinal Bacteroides (MIB) and the total eubacterial load (TL) were analyzed by quantitative RT-PCR and expressed as gene numbers per ng DNA. Data shown are representative for at least three independent experiments. (TIFF) [file pone.0176144.s001.tiff]

**A****Naive****d7****SPF**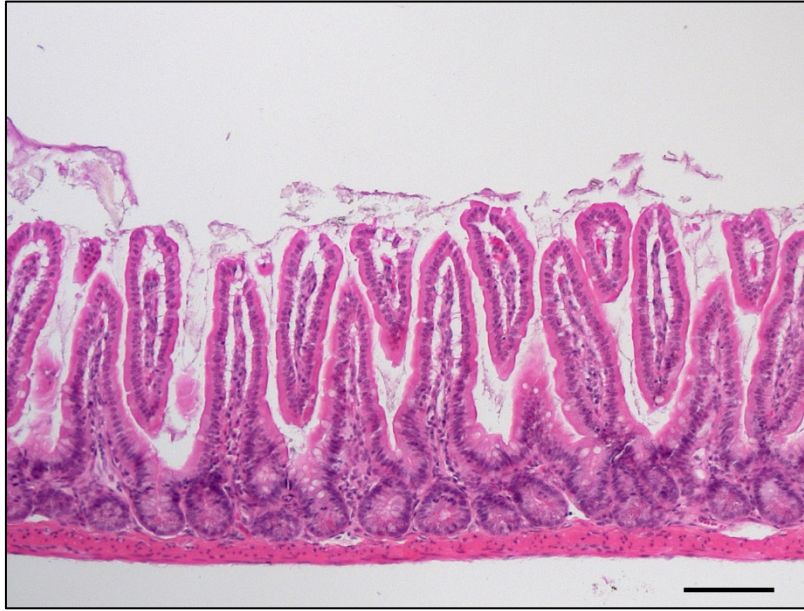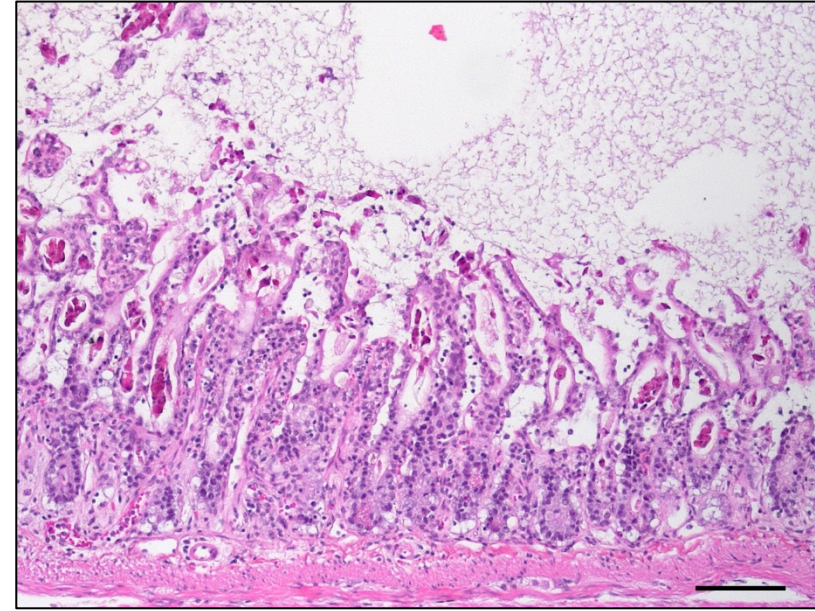**HMA**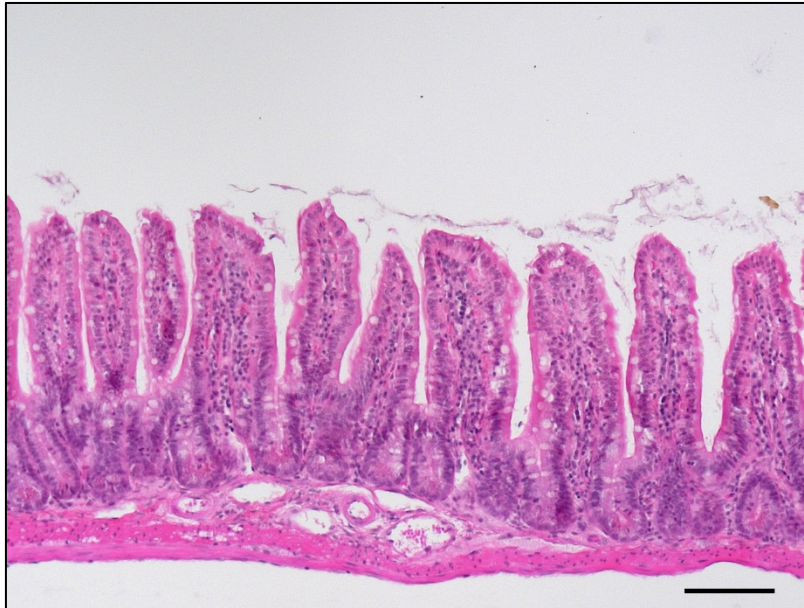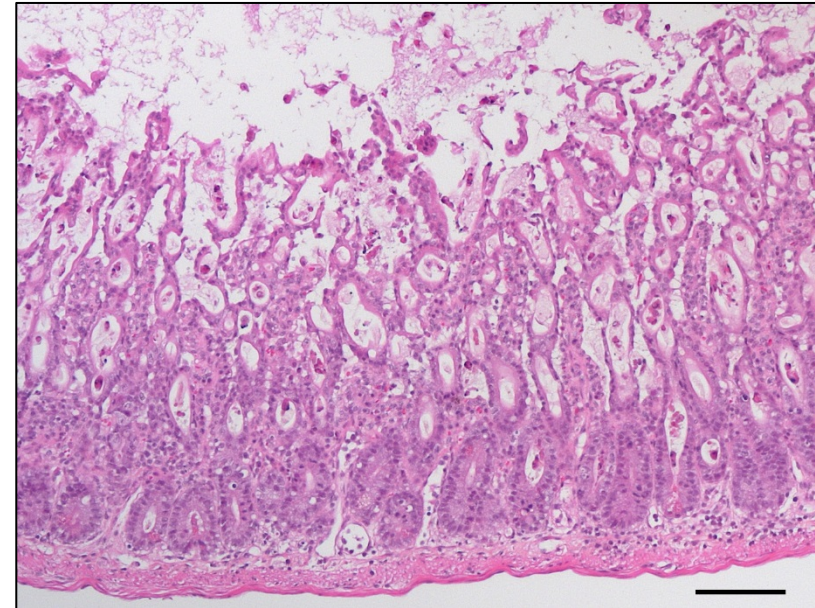

**B**

**Naive**

**d7**

**SPF**

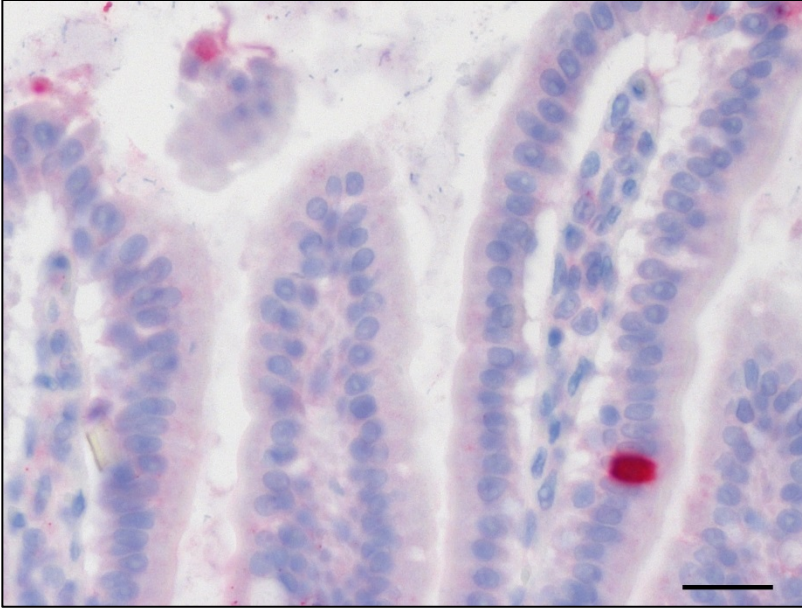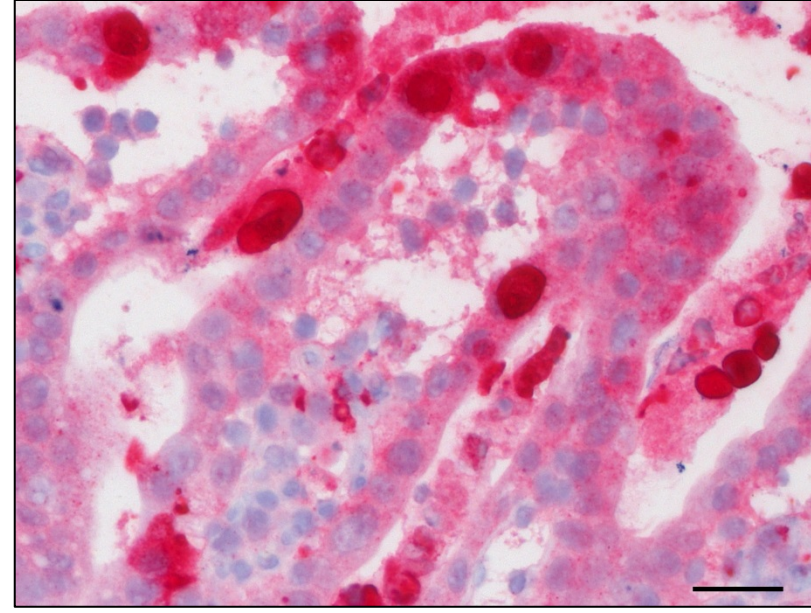

**HMA**

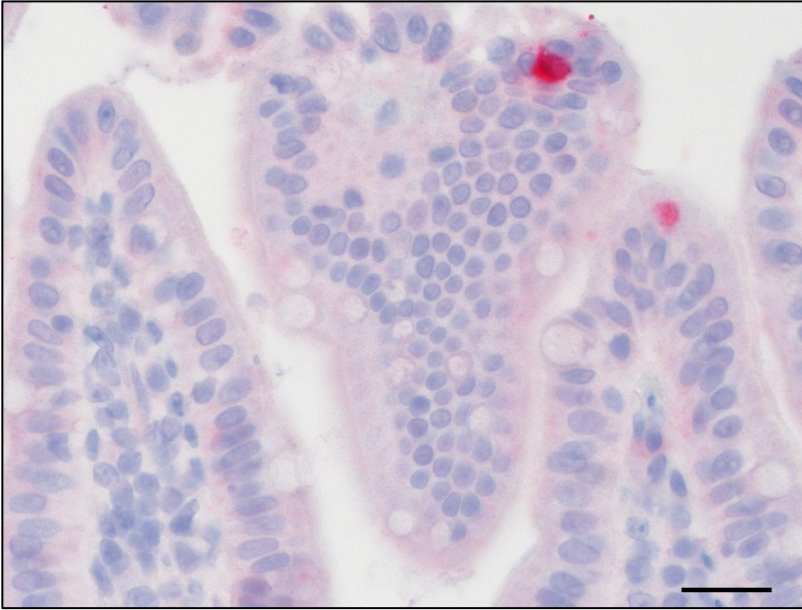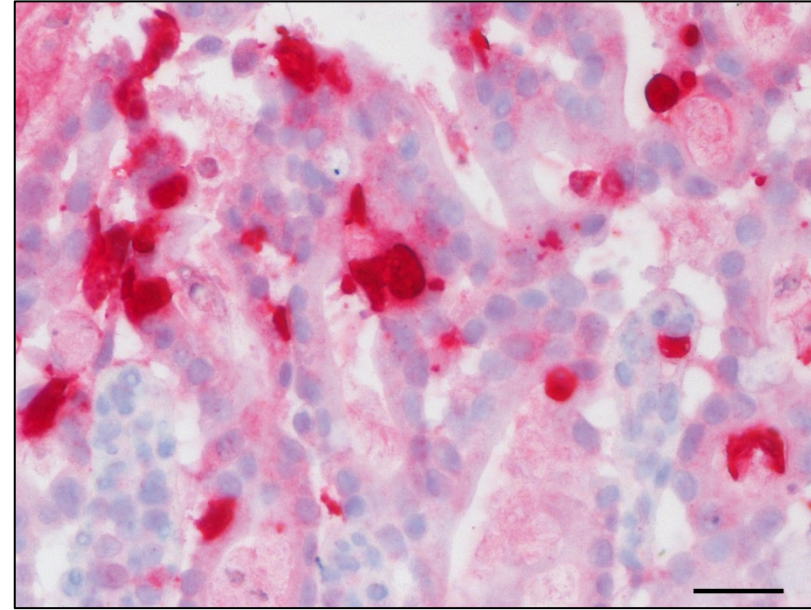

**C**

**Naive**

**d7**

**SPF**

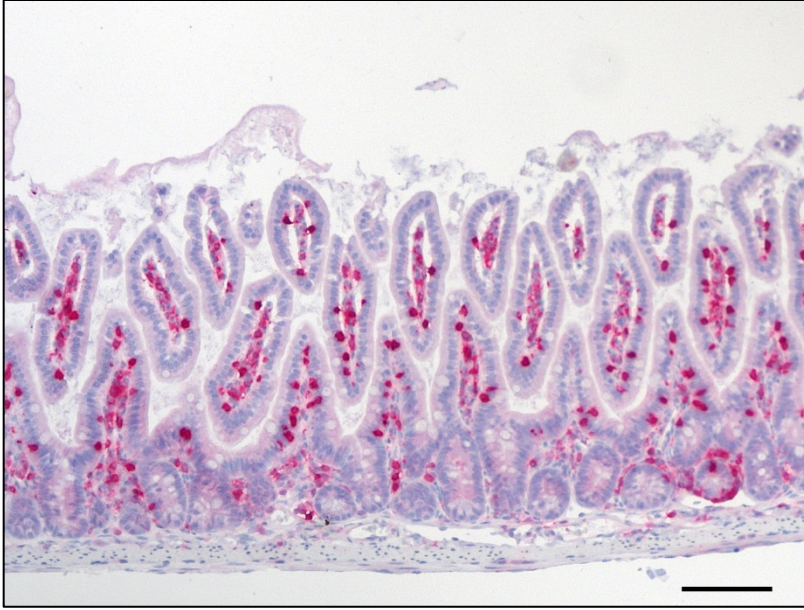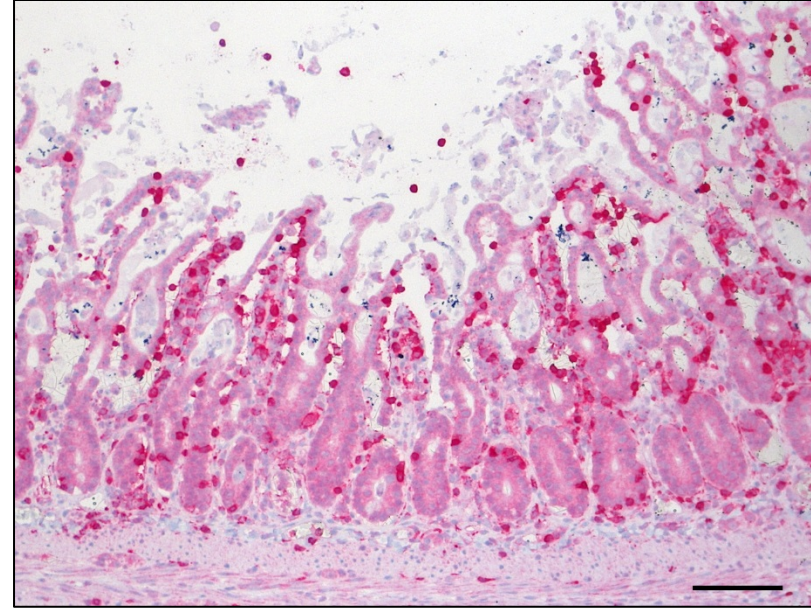

**HMA**

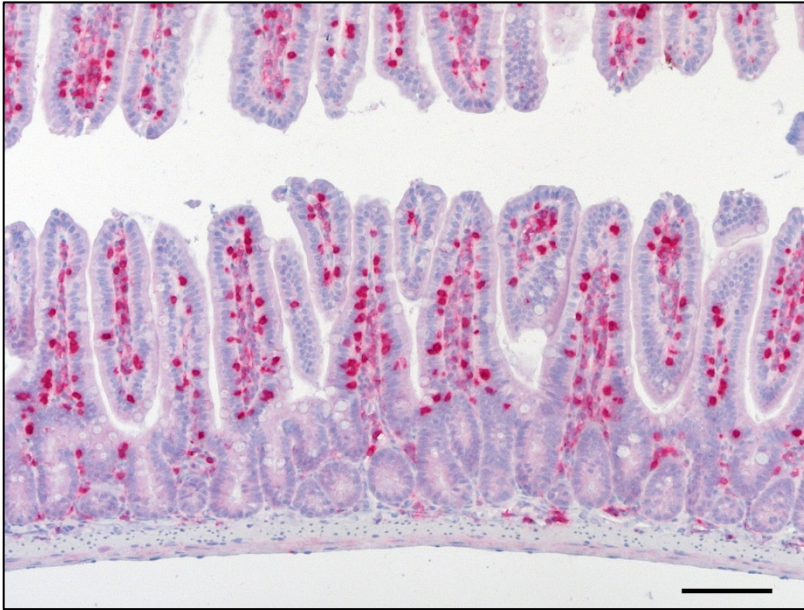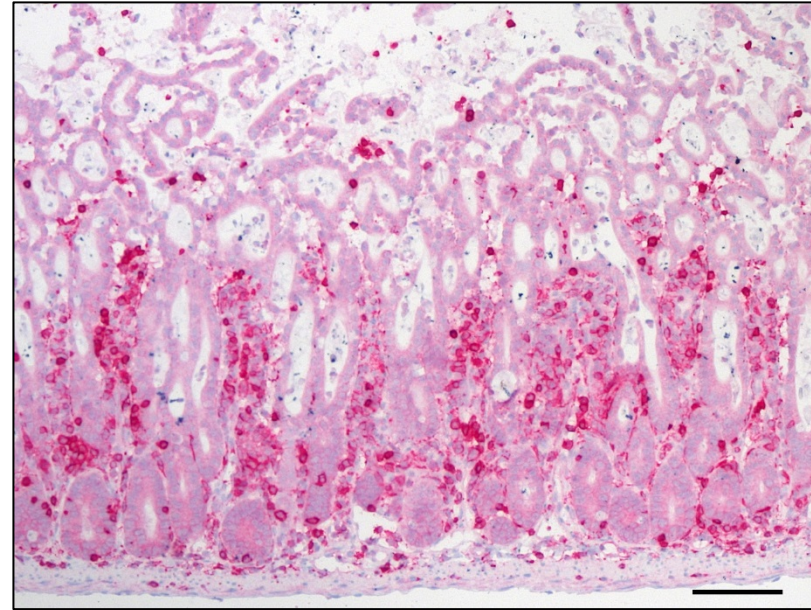

**D**

**Naive**

**d7**

**SPF**

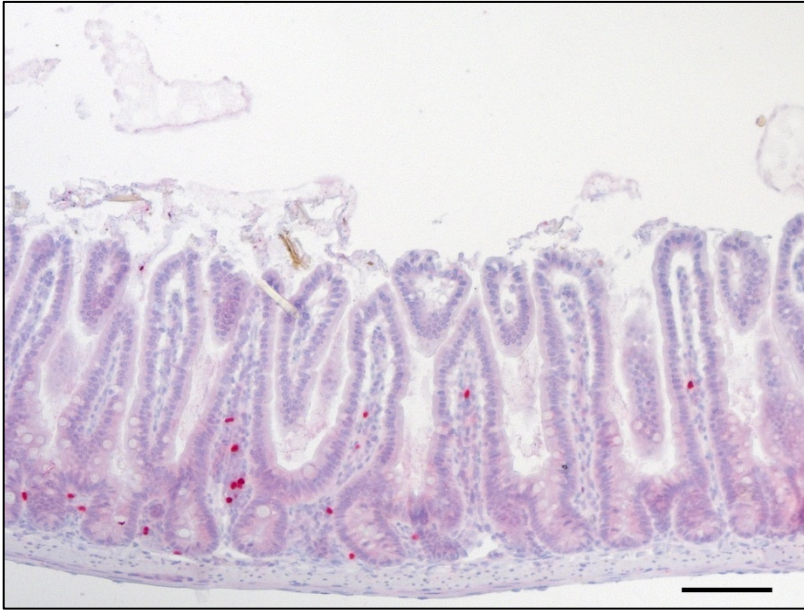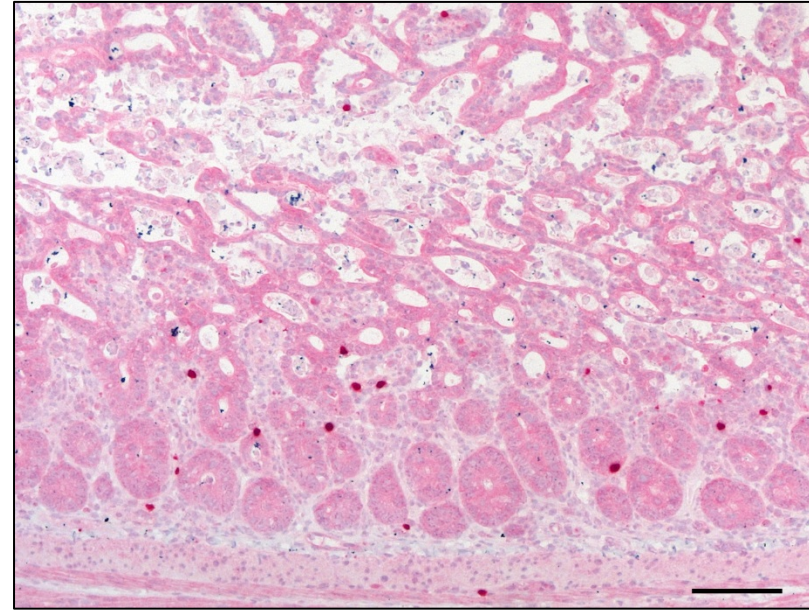

**HMA**

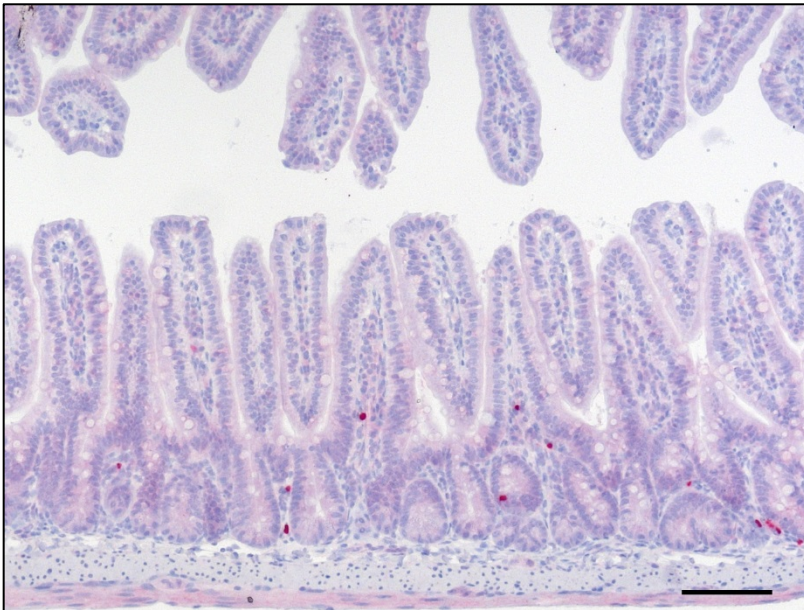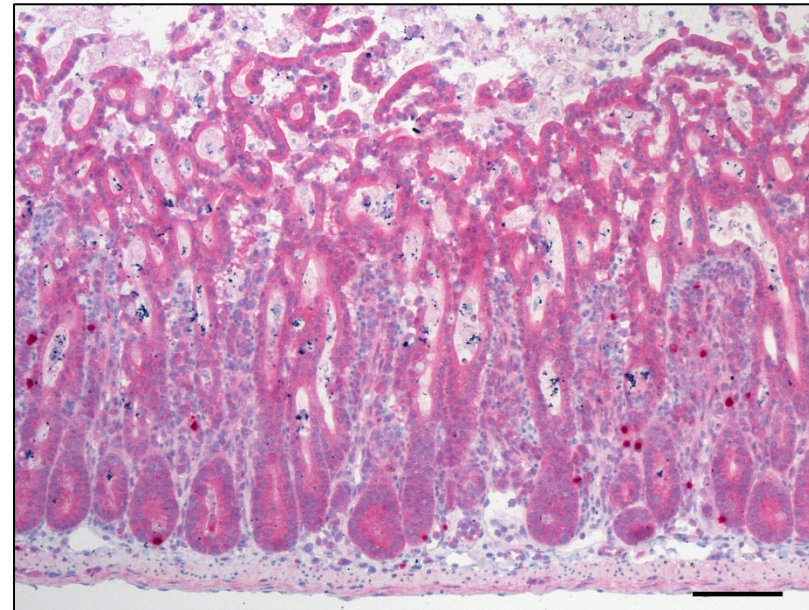

**M**

**Naive**

**d7**

**SPF**

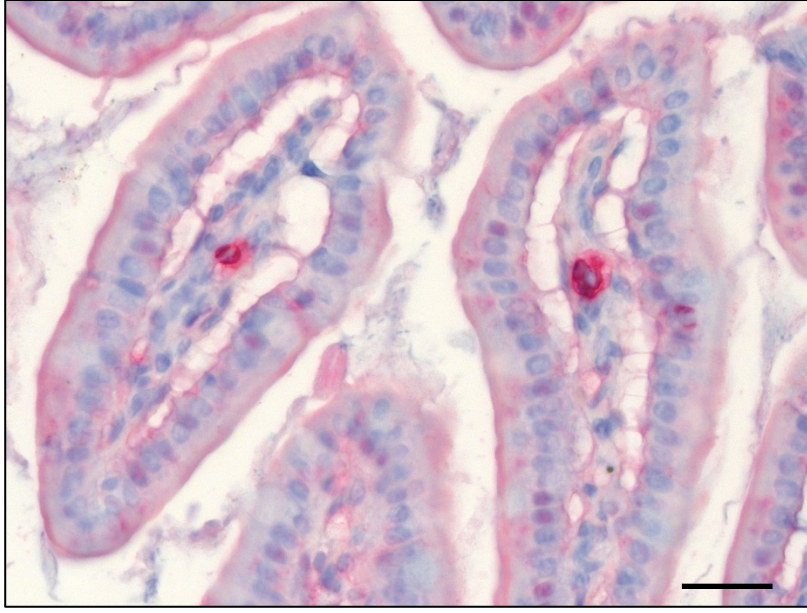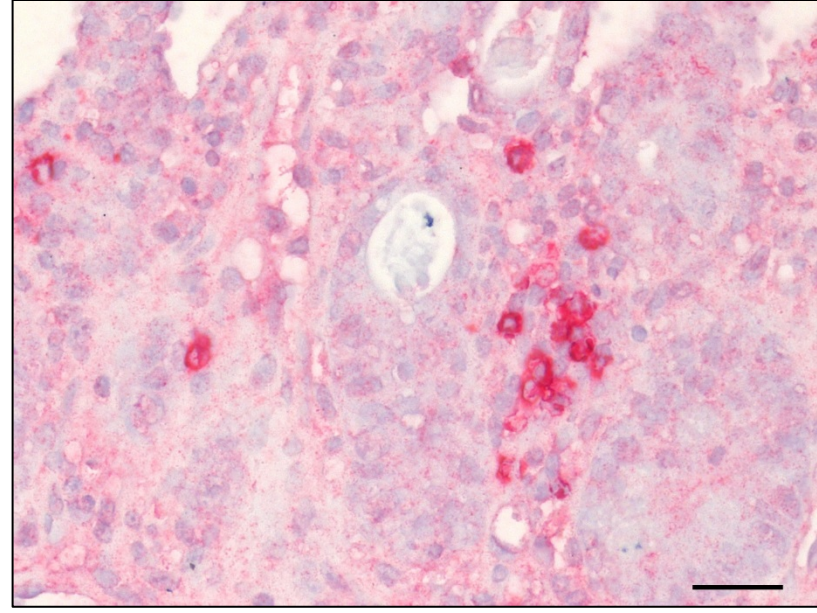

**HMA**

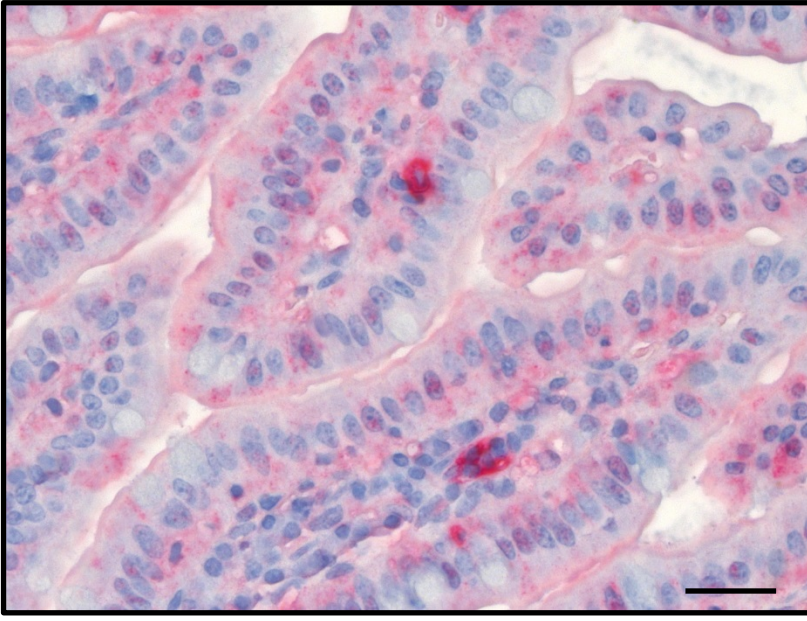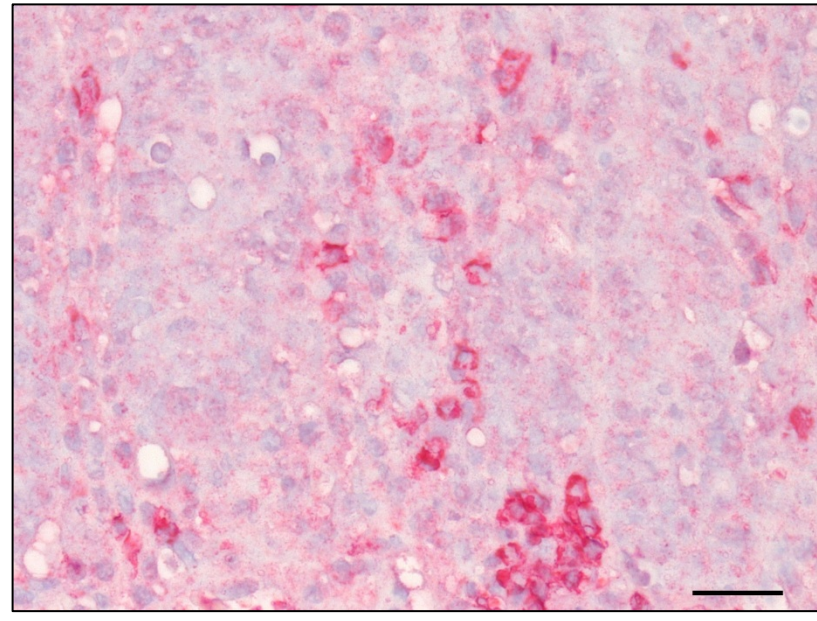

**F**

**Naive**

**d7**

**SPF**

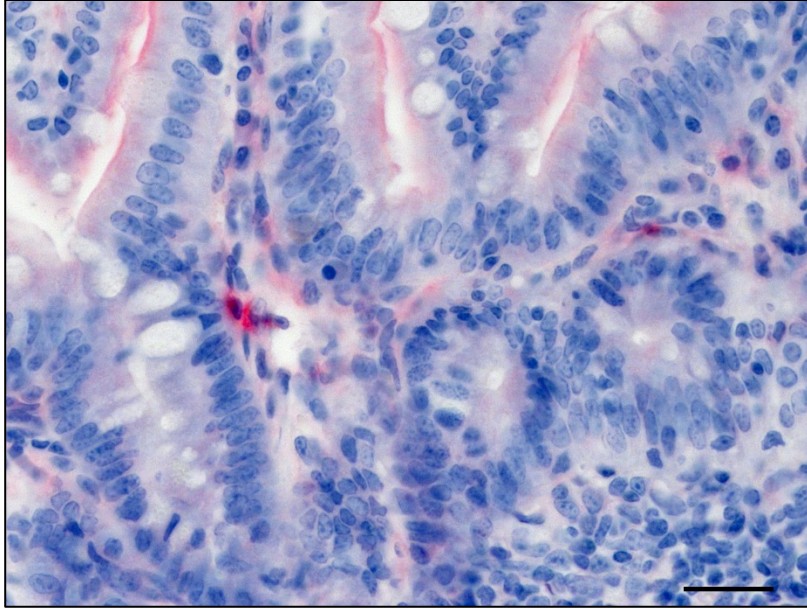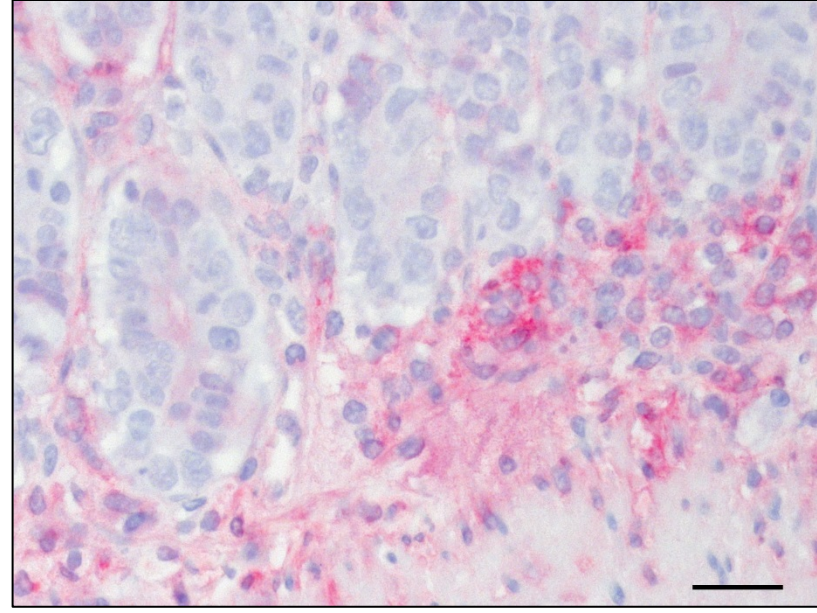

**HMA**

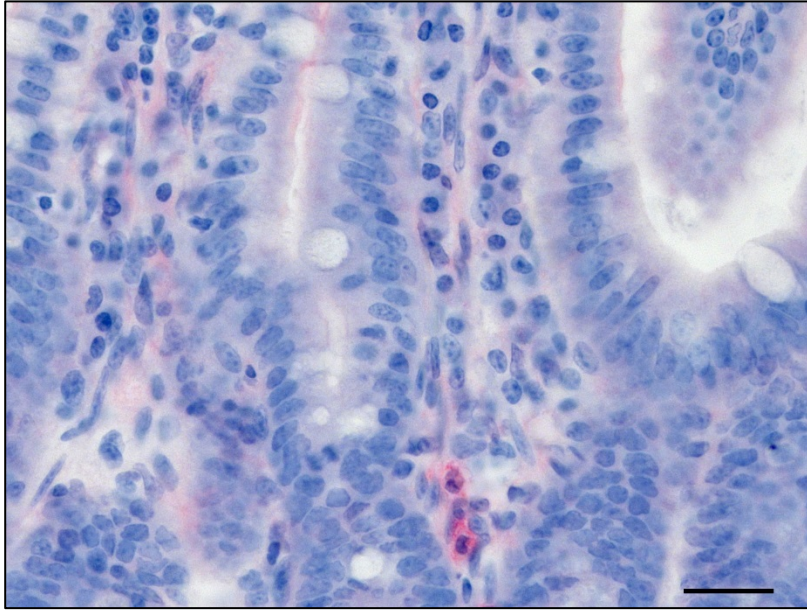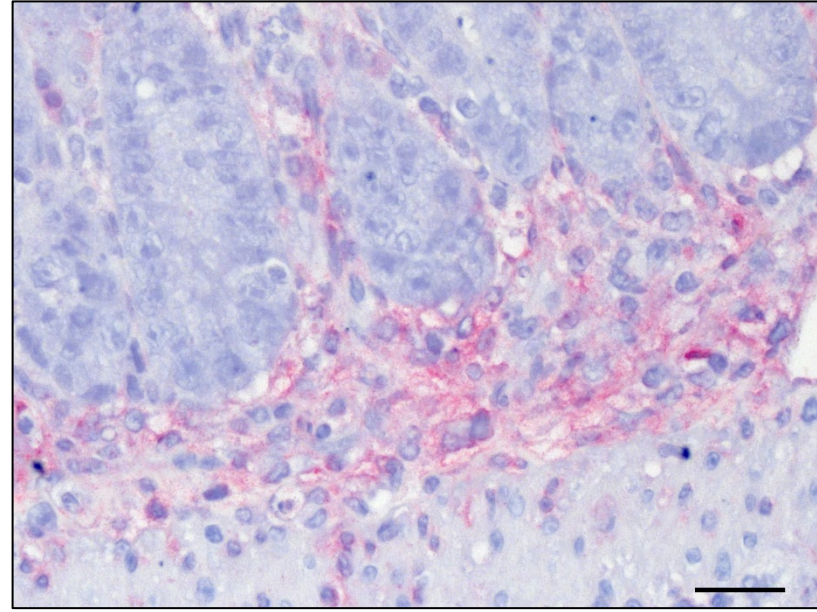

Supplement: S2 Fig — Human microbiota associated (hma) and conventionally colonized (SPF) mice were perorally infected with T. gondii strain ME49 to induce acute ileitis. Noninfected mice served as respective naive controls. Small intestinal immunohistopathological changes were assessed at day (d) 7 following ileitis induction in ileal paraffin sections stained with (A) hematoxylin & eosin or antibodies against (B) caspase-3, (C) CD3, (D) FOXP3, (E) B220 or (F) F4/80. Representative photomicrographs from three independent experiments are shown (A, C, D: 100 x magnification, scale bars 100 μm; B, E, F: 400 x magnification, scale bars 20 μm). (PDF) [file pone.0176144.s002.pdf]
